# A refractory liver metastatic solid pseudopapillary neoplasm pancreas harbored *CTNNB1* mutation showed good response to celecoxib: A case report

**DOI:** 10.3389/fonc.2022.1022290

**Published:** 2022-10-28

**Authors:** Yu Shang, Yunkun Zhang, Evenki Pan, Peng Yang, Lingling Xu, Jinghua Sun

**Affiliations:** ^1^ Department of Medical Oncology, The Second Hospital of Dalian Medical University, Dalian, Liaoning, China; ^2^ Department of Medical, Nanjing Geneseeq Technology Inc., Nanjing, Jiangsu, China

**Keywords:** Solid pseudopapillary neoplasm (SPN), liver metastasis, celecoxib, pancreas, CTNNB1 mutation

## Abstract

Solid pseudopapillary neoplasm (SPN) of the pancreas is rare relatively low-grade malignant neoplasm and metastasis rarely. Surgical resection is the primary treatment option for primary and metastatic lesions of SPN, and chemotherapy is often ineffective in non-operable SPNs. SPNs are characterized by the presence of somatic *CTNNB1* exon 3 mutations, leading to the activation of Wnt/β-catenin/Cox-2 signal pathway. Here, we firstly report that a refractory liver metastatic pancreatic SPN patient after the failure of multi-line chemotherapies benefited from the Cox-2 selective inhibitor (Celecoxib) based on *CTNNB1* D32V mutation detected by next-generation sequencing (NGS), achieving a more than 22-month progression-free survival without any adverse events. Our case provides a potential treatment option for liver metastatic SPN patients with *CTNNB1* mutations and highlights the application of NGS for the better treatment decision making.

## Introduction

Solid pseudopapillary neoplasm (SPN) of the pancreas is a rare and relatively low-grade malignant tumor, representing 1%-2% of pancreatic neoplasms, that occurs predominantly in young women (87%-90% of cases with average age of 20 to 30 years) (1–3). Most cases of SPN display an indolent behavior and commonly managed surgically with generally good prognosis, except for approximately 5%-20% of SPN patients with invasion and metastasis (4). SPN metastasis is very rare, which is reported in ~5% of cases, and liver is a common metastatic site (5, 6). In previous studies, increased tumor size, tumor capsular, pancreatic parenchymal invasions, synchronous metastasis and lymphovascular invasion were regarded as risk factors in association with SPN recurrence, and synchronous distant metastasis, lymphovascular invasion and increased tumor size were considered as worse prognostic factors for recurrence in patients with SPN (7–9).

SPNs have low genomic complexity and are characterized by the invariable presence of *CTNNB1* (a gene that encodes β-catenin) mutations with consequent activation of the Wnt signaling in over 90% of SPN cases (2). Currently, there is no consensus on effective systemic treatments for metastatic SPNs as this tumor is usually not sensitive to chemotherapy (10). Data on the inhibitor of the Wnt pathway for the treatment of SPN are limited, but the inhibitor may be an intuitive therapeutic option for this disease. Herein, we firstly report a refractory liver metastatic pancreatic SPN case who responded well to a selective cox-2 inhibitor (Celecoxib) monotherapy, with a 22-month progression-free survival (PFS), based on *CTNNB1* D32V hotspot mutation detected by NGS.

## Case report

**Figure 1:**
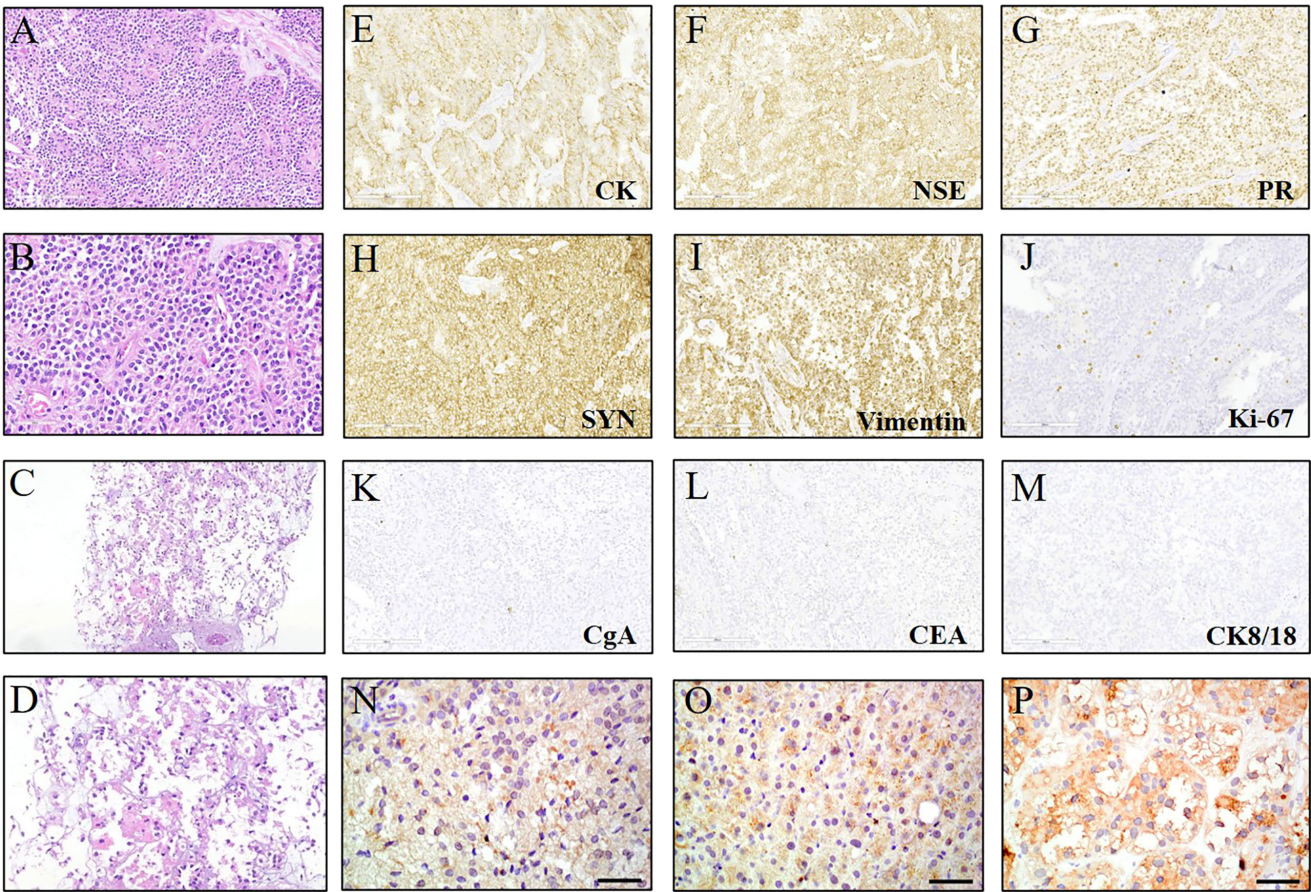
Hematoxylin & eosin (HE) and immunohistochemical (IHC) staining for the lesions. **(A, B)** HE staining (200× and 400×) of the primary solid pseudopapillary neoplasm (SPN) of the pancreas. **(C, D)** HE staining (100× and 200×) of the liver metastases at first discovery. **(E-M)** IHC examinations (200×) of the SPN which was positive for cytokeratin (CK), neuron-specific enolase (NSE), progesterone receptor (PR), synaptophysin (Syn) and Vimentin. The ki67 index was ≤5%, whereas negative for chromogranin A (CgA), carcinoembryonic antigen (CEA), cytokeratins 8 and 18 (CK8/18). **(N-P)** IHC staining (400×) of the primary pancreatic tissue and liver lesions showed the high expression of β-catenin.

A 45-year-old Chinese female presented with non-specific upper abdominal pain in August 2013. Computed tomography (CT) scan revealed a mass in the tail of the pancreas, which was removed by distal pancreatectomy with splenectomy. The pathology showed that the pancreatic tumor (5.5×6×4 cm) was composed of pleomorphic cells and polygonal cells that are either lamellar or arranged around a thin fibrovascular axis (**Figures 1A, B**). Immunohistochemistry analysis showed positive staining for cytokeratin (CK), neuron-specific enolase (NSE), progesterone receptor (PR), synaptophysin (Syn) and Vimentin. The Ki67 index was 3%, whereas negative staining for chromogranin A (CgA), carcinoembryonic antigen (CEA), cytokeratins 8 and 18 (CK8/18) (**Figures 1E-M**). Based on histological and immunohistochemical (IHC) results, she was diagnosed with SPN of the pancreas without metastasis.

**Figure 2:**
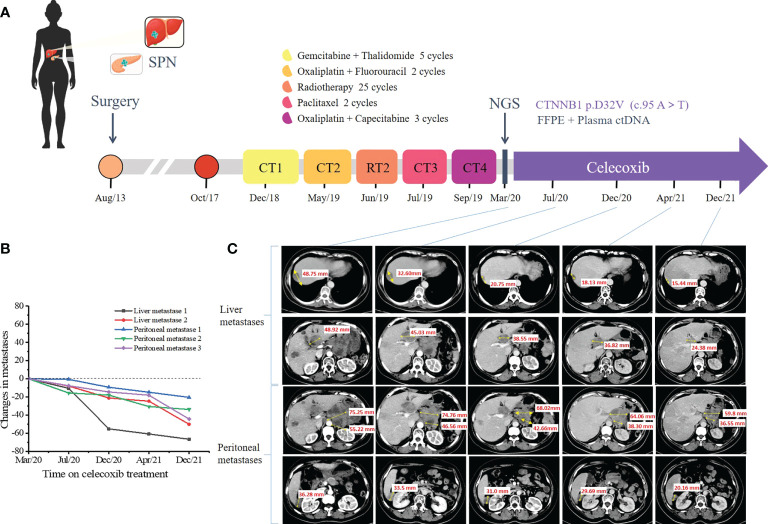
Representative clinical images during the treatment course. **(A)** Disease time line showed the various treatment received by the patient. **(B)** Scatter plot illustrates changes in liver metastases and peritoneal metastases. Computed tomography (CT) scan data at the first time to use celecoxib is chosen as time 0. **(C)** Computed tomography scan showed the multiple metastatic lesions in the liver and abdominal cavity under the celecoxib monotherapy. Tumor lesions were pointed with yellow arrows. (SPN, solid pseudopapillary neoplasm; CT, chemotherapy; RT, radiotherapy; NGC, next-generation sequencing).

During a follow-up of 5.1 years, CT scan and a liver biopsy confirmed the diagnosis of liver metastases (**Figures 1C, D**) and peritoneal metastases from SPN. In December 2018, she was treated with gemcitabine and thalidomide as first-line chemotherapy for 5 cycles (**Figure 2A**). Efficacy was evaluated based on CT scan alone and the follow-up interval was near 3 months. In May 2019, the tumors were evaluated as PD again, which led to second-line chemotherapy involving oxaliplatin and fluorouracil for 2 cycles and radiotherapy for 25 times (**Figure 2A**). Three months later, the CT scan showed progressive tumors in liver and abdominal cavity, so paclitaxel monotherapy was prescribed as third-line chemotherapy (**Figure 2A**). Unfortunately, in September 2019, the CT identified PD, the strategy switched to XELOX (capecitabine plus oxaliplatin) (**Figure 2A**). Six months later, the CT identified PD in liver and abdominal cavity.

To further search for a more efficient therapeutic strategy, genomic DNA was isolated from formalin-fixed paraffin-embedded (FFPE) tissues of the primary pancreatic lesions and liver lesions and circulating tumor DNA (ctDNA) from plasma for targeted next-generation sequencing (NGS) in March 2020. The targeted NGS of 425 cancer-related genes (**Supplementary Table S1**) was performed in Nanjing Geneseeq Technology Inc. approved by College of American Pathologists (CAP) and Clinical Laboratory Improvement Amendments (CLIA). A *CTNNB1* D32V (c. 95A>T) mutation was revealed at a mutant allele frequency (MAF) of 0.4% in plasma, 42.2% in pancreatic tissue and 14.3% in liver tissue. The detailed results of genetic alterations are shown in **Supplementary Table S2**. We further verified the high expression of β-catenin in primary pancreatic tissue and liver lesions by IHC staining (**Figures 1N–P**). In March 2020, the patient subjected to the treatment of celecoxib (200mg qd), which is a specific Cox-2 inhibitor. Surprisingly, lesions in pancreas, liver and abdominal cavity showed significant partial response (PR) 4 months later in July 2020. Therefore, the regimen was continued and the CT was performed to assess the tumor (**Figures 2B, C**). Until December 2021, the patient still achieved PR, with a 22-month progression-free survival (PFS) and continue to benefit from celecoxib monotherapy without any adverse events.

## Discussion

*CTNNB1* exon 3 gain-of function mutation is a characteristic molecular alteration in SPN (approximately 90% of cases) (4). Hot mutations that occur mainly at or around the glycogen synthase kinase-3beta (GSK-3β) phosphorylation site, interfere with normal phosphorylation of GSK-3-β and subsequent ubiquitin-mediated β-catenin degradation. β-catenin transfers into the nucleus and forms active complexes with intranuclear T-cell factor/lymphoid-enhancer factor (Tcf/Lef) family of DNA-binding proteins to regulate target gene expression, resulting int nuclear expression of β-catenin. The complex can activate the transcription of oncogenes such as *MYC* and *CCND1* and activate the Wnt/β-catenin signaling pathway (11). In addition, loss of cytoplasmic β-catenin protein in the cell adhesion complex results in instability of the complex, and loss of E-cadherin in cell membrane and cell cohesiveness, which eventually show a pseudopapillary pattern (11). Previous studies also have shown that *CTNNB1* mutations gene provoke constitutive accumulation of nuclear β-catenin express higher levels of some of Wnt pathway components such as LEF1, AXIN2, and RNF43 (2) and Cox-2/PGE2 pathway (12), which promote tumor growth.

Cox-2, a key enzyme required for the conversion of arachidonic acid to prostaglandins, plays an important role in the promotion of carcinogenesis, invasiveness, and angiogenesis (12). Previous studies have suggested a role for Wnt/β-catenin signaling during the onset and/or development of various types of cancer *via* modulating the expression of the Cox-2 gene (13–15). Felipe et al. demonstrated that Wnt/β-catenin components are involved in the transcriptional regulation of the Cox-2 gene (12). Thus, Cox-2 expression increases as β-catenin target gene after its nucleation, further, Cox-2 activation in turn continues to promote β-catenin nucleation, and form a malignant positive-feedback loop crosstalk that drives SPN development (13, 14). Therefore, targeting inhibition of Cox-2 may be a breakthrough in the treatment of SPN.

Previous studies demonstrated that the resection of metastatic lesion is the primary treatment option for SPN patients with liver metastasis (16). However, the surgery may not be performed in patients with extensive liver metastasis. SPN is also insensitive to chemotherapy and makes the choice of systemic treatment options difficult (2, 10). Wang et al. reported that a metastatic pancreatic SPN case maintained stable disease by the targeted agents sunitinib and everolimus based on *PTEN* and *CTNNB1* mutations (17). However, it was reported that the use of sunitinib and everolimus was associated with an increased risk of adverse events (18, 19). Celecoxib, a Cox-2 selective inhibitor, has been proven to be effective in patients with desmoid tumors with *CTNNB1* mutations (20, 21). In the present case, the *CTNNB1* D32V mutation, which was also the exon 3 of *CTNNB1* mutation, was detected by targeted NGS in the pancreatic and liver tissue and the plasma ctDNA collected after the failure of multi-line chemotherapies and verified in primary pancreatic tissue and liver lesions by IHC staining. We then use celecoxib monotherapy for the patient, which led to the best PR in lesions of pancreas, liver and abdominal cavity (PFS = 22 months) and no adverse events. The limitation of the single-case presentation in this study should be noted. Thus, the efficacy and adverse events of the treatment with Cox-2 selective inhibitor (Celecoxib) must be further evaluated in larger cohorts. The missense mutation of *CTNNB1* (D32V) in this case might be a potential target of Cox-2 inhibitors, however, additional pre-clinical studies and additional clinical evidence are needed. Since the amount of tissue biopsy samples was limited that were not enough for underestimate the LEF1 and COX-2 status, their importance in exploring the mechanism of COX-2 selective inhibitor (Celecoxib) therapy in this patient with SPN should be further studied. Meanwhile, no comprehensive evaluation such as PET was performed during follow-up, this limitation should also be noted.

## Conclusion

In summary, we reported the first case of a refractory liver metastatic SPN patient with a *CTNNB1* D32V mutation, who received the treatment of celecoxib after the failure of multi-line chemotherapies and achieved a PFS of 22 months without any adverse events. This report provides a reliable treatment option for liver metastatic SPN patients with *CTNNB1* D32V mutations. Moreover, we also highlighted the importance of NGS for the better treatment decision making and the use of targeted therapy. Due to the single-case presentation in this study, more clinical evidence is needed to be further studied.

## Data availability statement

The original contributions presented in the study are included in the article/**Supplementary Material**. Further inquiries can be directed to the corresponding authors.

## Ethics statement

The studies involving human participants were reviewed and approved by the Ethics Committee of The Second Hospital of Dalian Medical University. The patients/participants provided their written informed consent to participate in this study. Written informed consent was obtained from the individual(s), and minor(s)’ legal guardian/next of kin, for the publication of any potentially identifiable images or data included in this article.

## Author contributions

All authors contributed to data analysis and drafting or revising of the manuscript. All authors agreed on the journal to which the article is submitted, provided final approval of the manuscript version to be published, and agreed to be accountable for all aspects of the study. All authors contributed to the article and approved the submitted version.

## Conflict of interest

Authors EP and PY are employed by Nanjing Geneseeq Technology Inc.

The remaining authors declare that the research was conducted in the absence of any commercial or financial relationships that could be constructed as a potential conflict of interest.

## Publisher’s note

All claims expressed in this article are solely those of the authors and do not necessarily represent those of their affiliated organizations, or those of the publisher, the editors and the reviewers. Any product that may be evaluated in this article, or claim that may be made by its manufacturer, is not guaranteed or endorsed by the publisher.
