# Supplementary material for: A refractory liver metastatic solid pseudopapillary neoplasm pancreas harbored CTNNB1 mutation showed good response to celecoxib: A case report
Source: Front Oncol. 2022 Oct 28;12:1022290. doi: 10.3389/fonc.2022.1022290 (PMC9650638; doi:10.3389/fonc.2022.1022290)
Supplement: Supplementary file 1 [file DataSheet_1.docx]

**Supplementary Table S1.** Gene list of 425 cancer-related genes.

| *ABCB1 (MDR1), ABCB4, ABCC2 (MRP2), ADH1A, ADH1B, ADH1C, AIP, AKT1, AKT2, AKT3, ALDH2, ALK, AMER1, APC, AR, ARAF, ARID1A, ARID1B, ARID2, ARID5B, ASCL4, ASXL1, ATF1, ATIC, ATM, ATR, ATRX, AURKA, AURKB, AXIN2, AXL, B2M, BAD, BAI3, BAK1, BAP1, BARD1, BAX, BCL2, BCL2L11 (BIM), BCR, BIRC3, BLM, BMPR1A, BRAF, BRCA1, BRCA2, BRD4, BRIP1, BTG2, BTK, BUB1B, CLLORF30, CASP8, CBL, CBLB, CCND1, CCNE1, CD274 (PD-L1), CD74, CDA, CDC73, CDH1, CDK10, CDK12, CDK4, CDK6, CDK8, CDKN1A, CDKN1B, CDKN1C, CDKN2A, CDKN2B, CDKN2C, CEBPA, CEP57, CHD4, CHEK1, CHEK2, CREBBP, CRKL, CSF1R, CTCF, CTLA4, CTNNB1, CUL3, CUX1, CXCR4, CYLD, CYP19A1, CYP2A13, CYP2A6, CYP2A7, CYP2B6*6, CYP2C19*2, CYP2C9*3, CYP2D6, CYP3A4*4, CYP3A5, DAXX, DDR2, DENND1A, DHFR, DICER1, DLL3, DNMT3A, DPYD, DUSP2, EGFR, EML4, EP300, EPAS1, EPCAM, EPHA2, EPHA3, EPHA5, EPHB2, ERBB2 (HER2), ERBB2IP, ERBB3, ERBB4, ERCC1, ERCC2, ERCC3, ERCC4, ERCC5, ESR1, ETV1, ETV4, ETV6, EWSR1, EXT1, EXT2, EZH2, FANCA, FANCC, FANCD2, FANCE, FANCF, FANCG, FANCI, FANCL, FANCM, FAT1, FBXW7, FGF19, FGFR1, FGFR2, FGFR3, FGFR4, FH, FLCN, FLT1 (VEGFR1), FLT3, FLT4, FOXA1, FOXP1, FRG1, GATA1, GATA2, GATA3, GATA4, GATA6, GNA11, GNAQ, GNAS, GRIN2A, GRM3, GRM8, GSTM1, GSTM4, GSTM5, GSTP1, GSTT1, HDAC2, HDAC9, HGF, HLA-A, HNF1A, HNF1B, HRAS, HSD3B1, IDH1, IDH2, IFNG, IFNGR1, IGF1R, IGF2, IKBKE, IKZF1, IL7R, INPP4B, IRF2, JAK1, JAK2, JAK3, JARID2, JUN, KDM5A, KDM6A, KDR (VEGFR2), KEAP1, KIF1B, KIF5B, KIT, KITLG, KLLN, KMT2A (MLL), KMT2B, KMT2C, KMT2D, KRAS, LHCGR, LMO1, LRP1B, LYN, LZTR1, MAP2K1 (MEK1), MAP2K2 (MEK2), MAP2K4, MAP3K1, MAP3K4, MAP4K3, MAX, MCL1, MDM2, MDM4, MECOM, MED12, MEF2B, MEN1, MET, MGMT, MITF, MLH1, MLH3, MLLT1, MLLT3, MLLT4, MPL, MRE11A, MSH2, MSH6, MTHFR, MTOR, MUTYH, MYC, MYC, MYCN, MYD88, MYH9, NAT1, NBN NCOR1, NF1, NF2, NFE2L2, NFKBIA, NKX2-1, NKX2-4, NOTCH1, NOTCH2, NOTCH3, NPM1, NQO1, NRAS, NRG1, NSD1, NTRK1, NTRK2, NTRK3, PAK3, PALB2, PALLD, PARK2, PARP1, PARP2, PAX5, PBRM1, PDCD1 (PD1), PDCD1LG2 (PD-L2), PDE11A, PDGFRA, PDGFRB, PDK1, PGR, PHOX2B, PIK3C3, PIK3CA, PIK3R1, PIK3R2, PKHD1, PLAG1, PLK1, PMS1, PMS2, POLD1, POLD3, POLE, POLH, POT1, PPARD, PPP2R1A, PRDM1, PRF1, PRKACA, PRKACG, PRKAR1A, PRKCI, PRKDC, PRSS1, PRSS3, PTCH1, PTEN, PTK2, PTPN11, PTPN13, PTPRD, QKI, RAC1, RAC3, RAD50, RAD51, RAD51B, RAD51C, RAD51D, RAD54L, RAF1, RARA, RARG, RASGEF1A, RB1, RECQL4, RELN, RET, RHOA, RICTOR, RNF43, ROS1, RPTOR, RRM1, RUNX1, RUNX1T1, SBDS, SDC4, SDHA, SDHB, SDHC, SDHD, SEPT9, SETBP1, SETD2, SF3B1, SGK1, SLC34A2, SLC3A2, SLC7A8, SMAD2, SMAD3, SMAD4, SMAD7, SMARCA4, SMARCB1, SMO, SOS1, SOX1, SOX14, SOX2, SOX21, SPOP, SPRY4, SRC, SRY, STAG2, STAT3, STK11, STMN1, STT3A, SUFU, TAP1, TAP2, TEK, TEKT4, TERC, TERT, TET2, TGFBR2, THADA, TMEM127, TMPRSS2, TNFAIP3, TNFRSF11A, TNFRSF14, TNFRSF19, TNFSF11, TOP1, OP2A, TP53, TP63, TPMT, TSC1, TSC2, TSHR, TTF1, TUBB3, TUBB4A, TUBB4B, TUBB6, TYMS, U2AF1, UGT1A1, VAMP2, VEGFA, VHL, WAS, WISP3, WRN, WT1, XPA, XPC, XRCC1, YAP1, ZNF2, ZNF217, ZNF703* |
| --- |

**Supplementary Table S2.** The allele frequencies of genetic alterations detected by targeted NGS Sequencing in different sample types.

| Gene | Alteration | sample type | | |
| --- | --- | --- | --- | --- |
|  |  | Plasma ctDNA | pancreatic tissue (FFPE) | liver tissue (FFPE) |
| *CTNNB1* | D32V | 0.4% | 42.2% | 14.3% |
| *ERBB3* | R453H | - | 5.0% | - |
| *GATA1* | G128Afs*9 | 0.4% | - | - |
| *PLCB4* | T238M | 19.7% | 36.7% | 41.3% |
| *RB1* | RB1~DIAPH3 fusion | - | 27.5% | 6.7% |
| *RECQL4* | H725Q | - | 3.4% | - |
| *IDH2* | T435M | - | 1.0% | - |

FFPE: formalin-fixed, paraffin-embedded; “-”: not detected; ctDNA: circulating tumor DNA.
